# 

**Published:** 1849-11

**Authors:** 


No. VI. NOVEMBER. Vol. V.

### BUFFALO

### MEDICAL JOURNAL

AND

### MONTHLY REVIEW

OF

### MEDICAL AND SURGICAL SCIENCE.

EDITED BY AUSTIN FLINT, M. D.

BUFFALO:
PUBLISHED BY JEWETT, THOMAS & CO.
Commercial Advertiser Buildings.

1849.

